# The emerging roles of leukocyte cell-derived chemotaxin-2 in immune diseases: From mechanisms to therapeutic potential

**DOI:** 10.3389/fimmu.2023.1158083

**Published:** 2023-03-09

**Authors:** Ming-Hui Zhu, Yan-Jun Liu, Chang-Yun Li, Fan Tao, Guan-Jun Yang, Jiong Chen

**Affiliations:** ^1^ State Key Laboratory for Managing Biotic and Chemical Threats to the Quality and Safety of Agro-products, Ningbo University, Ningbo, Zhejiang, China; ^2^ Laboratory of Biochemistry and Molecular Biology, School of Marine Sciences, Ningbo University, Ningbo, China; ^3^ Key Laboratory of Aquacultural Biotechnology Ministry of Education, Ningbo University, Ningbo, China

**Keywords:** LECT2, LECT2 receptor, immune regulation, inflammation-related diseases, comparative immunology

## Abstract

Leukocyte cell-derived chemotaxin-2 (LECT2, also named ChM-II), initially identified as a chemokine mediating neutrophil migration, is a multifunctional secreted factor involved in diverse physiological and pathological processes. The high sequence similarity of LECT2 among different vertebrates makes it possible to explore its functions by using comparative biology. LECT2 is associated with many immune processes and immune-related diseases *via* its binding to cell surface receptors such as CD209a, Tie1, and Met in various cell types. In addition, the misfolding LECT2 leads to the amyloidosis of several crucial tissues (kidney, liver, and lung, etc.) by inducing the formation of insoluble fibrils. However, the mechanisms of LECT2-mediated diverse immune pathogenic conditions in various tissues remain to be fully elucidated due to the functional and signaling heterogeneity. Here, we provide a comprehensive summary of the structure, the “double-edged sword” function, and the extensive signaling pathways of LECT2 in immune diseases, as well as the potential applications of LECT2 in therapeutic interventions in preclinical or clinical trials. This review provides an integrated perspective on the current understanding of how LECT2 is associated with immune diseases, with the aim of facilitating the development of drugs or probes against LECT2 for the theranostics of immune-related diseases.

## 1 Introduction

LECT2 (leucocyte cell-derived chemotaxin 2) is a hormone-like protein that was originally identified as a chemokine mediating neutrophil migration (1). Subsequently, it has also been defined as chondromodulin II (CHM2 or ChM-II) due to its function in promoting chondrocyte proteoglycan synthesis and cartilage growth (2). In fact, as a member of chondromodulin family, although CHM2 shares lower sequence similarity with its family member CHM-1, both of them function as anti-angiogenic factors (3, 4). CHM2 suppresses angiogenesis by blocking VEGF165-VEGFR2 signaling in liver cancer (5) and reduces endothelial cell migration and tube formations by activating LECT2-Tie1 signaling in liver fibrosis (6).

LECT2 is also identified as a hepatokine. Hepatocyte-derived LECT2 not only regulates hepatocyte cells in an autocrine mode, but can also be secreted into the bloodstream to act on cells of other tissues in a paracrine way to modulate multiple metabolic homeostasis or disorders such as glucose metabolism, non-alcoholic fatty liver disease (NAFLD) (7, 8), alcohol-induced liver cirrhosis (9), obesity (10), diabetes (11, 12), and atherosclerosis (13). LECT2 is also positively correlated with diet-induced weight cycling in mice and humans, suggesting that LECT2 is a sensing hepatokine for nutritional regulation-mediated metabolic homeostasis and functions as an indicator for management of obesity in the clinic (14, 15).

Besides its soluble functioning as a cytokine or hepatokine, LECT2 also exists in the form of amyloidosis (aLECT2) and is involved in renal and hepatic amyloid lesions (16–18). aLECT2 has been found to deposit in vessels, interstitial, and glomeruli of renal biopsies (19–21). The mutation or genetic variations of LECT2 are responsible for the formation of aLECT2, while the misfolding LECT2, which leads to insoluble fibrils aggregated in cells and tissues, might be the potential pathogenesis of LECT2-mediated amyloidosis (19, 20). However, the specific role of aLECT2 in amyloidosis is still unclear, and there is also a disputation about whether this protein is feasible as a diagnosis and treatment for aLECT2-mediated amyloidosis.

Furthermore, more recent research has revealed the link between LECT2 and the development of multiple immunological diseases such as sepsis (22–24), atherosclerosis (13, 25–27), osteoporosis (28, 29), arthritis (30–33), diabetes (10–12, 34), atopic dermatitis (35), and non-alcoholic steatohepatitis (NASH) (7, 8). Nonetheless, it is disease-dependent for the action mechanisms and signaling of LECT2. Thus, herein, we have analyzed the expression and roles of LECT2 and its ligand proteins in various inflammatory diseases to provide a comprehensive review that will help researchers examining these processes and determining the bioavailability of LECT2 in the future.

## 2 The molecular structure and tissue distribution of LECT2

**Figure 1:**
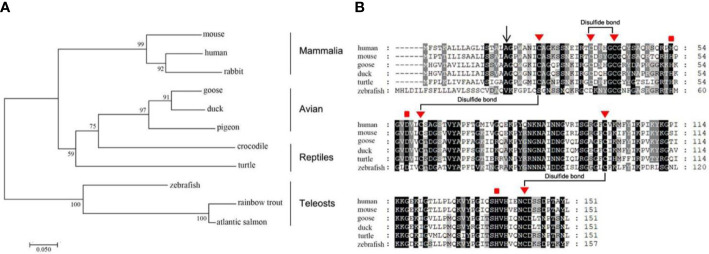
Analysis of the evolution and conservation of LECT2 in vertebrates. The phylogenetic analysis (neighbor-joining) **(A)** and multiple alignments **(B)** of the amino acid sequences of LECT2 in a variety of vertebrates. “▮” represents metal-binding sites.

The human LECT2 gene is located at chromosome 5q31.1–32 and consists of three introns and four exons (36). Its cDNA is 456 nucleotides (nts) in length, containing an open reading frame encoding a polypeptide of 151 amino acid (aa) residues with a calculated molecular weight of 16.39 kDa and a isoelectric point of 9.42 (36). The LECT2 protein is the only member of the zinc-dependent metalloendopeptidases M23 family in vertebrates, which contains a zinc ion as a cofactor and prefers peptides containing polyglycine residues (37, 38). The phylogenic analysis shows that LECT2 is highly conserved from teleosts to Mammalia (**Figure 1A**). All of them have a signal peptide, three conserved disulfide bonds, and three metal-binding sites (**Figure 1B**). These conserved sequences or sites are crucial for the functioning of LECT2, and they also provide the theoretical basis to explore the functions of LECT2 by comparative biology.

LECT2 is primarily produced in hepatocytes and mainly secreted to the bloodstream (16), but it is also found in other tissues or cells, such as macrophages (8, 13), parathyroid cells (39), adipocytes (10, 40), cerebral nerve cells (39), and vascular endothelial cells (6, 26).

## 3 LECT2-mediated signaling

**Figure 2:**
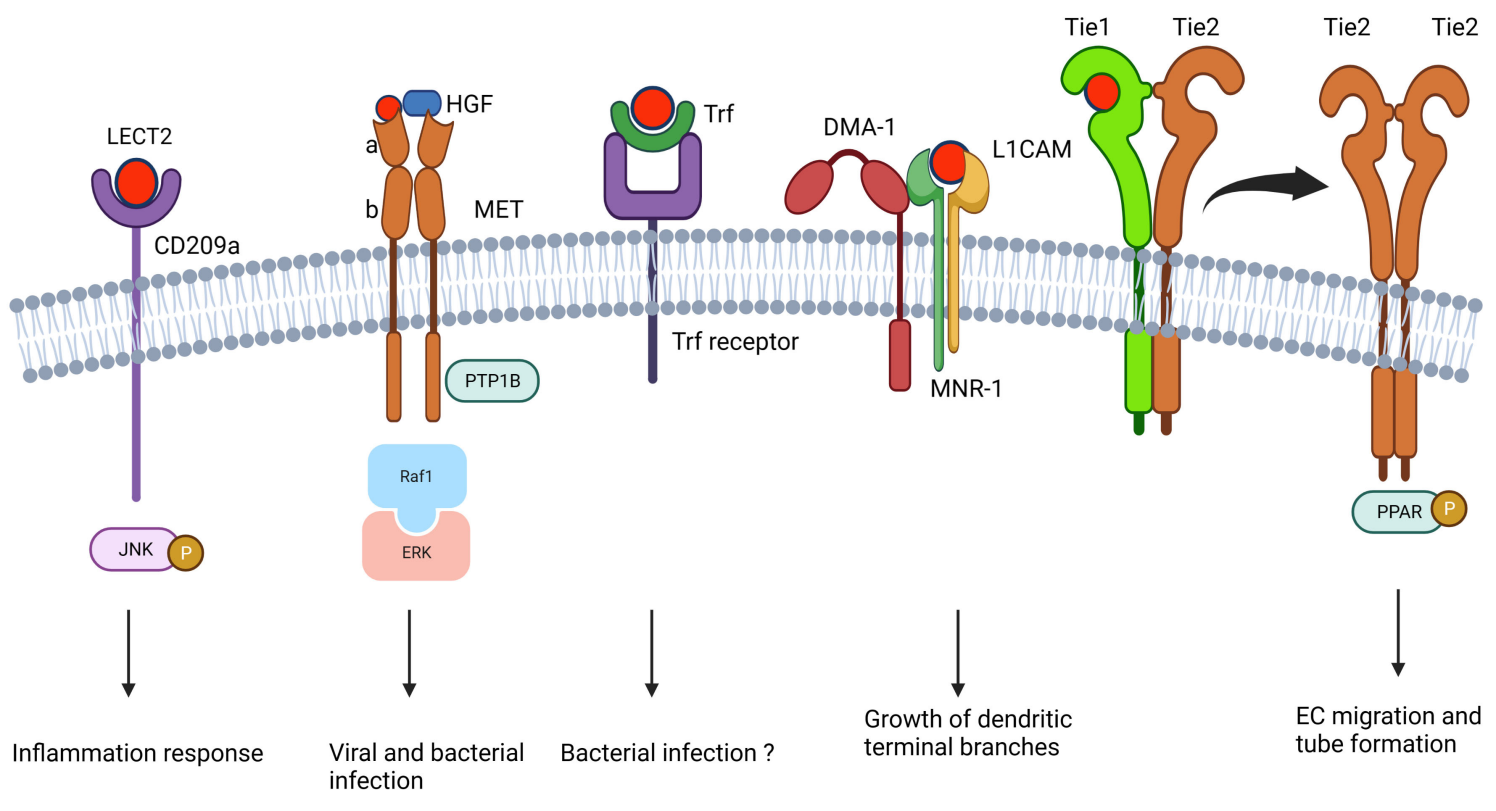
The reported LECT2-mediated signaling in the literature.

LECT2 exhibits its pleiotropic functions *via* its receptors, including CD209 antigen-like protein A (CD209a) (22, 41), tyrosine kinase with immunoglobulin-like and EGF-like domains 1 (Tie1) (6), MET (tyrosine protein kinase Met, also called c‐Met) (37, 42, 43), L1 cell adhesion molecule (L1CAM or SAX-7) (44), MNR-1 (44), and transferrin (Trf) (45) (**Figure 2**). CD209a is the first identified LECT2 receptor discovered by our group (41), and it contributes to enhancing the bacterial clearance ability of macrophages by phosphorylating the c-Jun N-terminal kinase (JNC) (**Figure 2A**) (22, 41). c-Met is the other receptor of LECT2, and the c-Met-LECT2 protein–protein interaction (PPI) impedes MET receptor activation to inhibit vascular invasion, metastasis, proliferation, and stemness of several cancers by antagonizing different cancer activation pathways (**Figure 2B**) (43, 46–48). In addition, Shirasaki et al. found that LECT2 functions as an anti-viral protein against lymphocytic choriomeningitis virus (LCMV) by binding to c-Met and thus competes with HGF-MET signaling (**Figure 2B**) (42). Tie1, a well-known angiopoietin receptor in many angiogenesis-related physiological and pathological processes (49), was also found to interact with LECT2. LECT2 promotes the dissociation of Tie1-Tie2 heterodimerization and the formation of Tie1-Tie2 homodimerization, which surpasses the invasion and metastasis of endothelial cells by activating PARP signaling (**Figure 2E**) (6). In *Caenorhabditis elegans*, muscle-secreted LECT-2 is an orthologue of vertebrate LECT2. It forms a multiprotein receptor–ligand complex with two skin transmembrane ligands, L1CAM and MNR-1, and a neuronal transmembrane receptor, DMA-1, which guides the growth of dendritic terminal branches (**Figure 2D**) (44). Trf is a glycoprotein with iron-binding and anti-microbial activity in vertebrates (**Figure 2C**) (50). Our group revealed that LECT2 interacts with Trf, and this interaction is highly conserved from fish to mouse (45).

## 4 The roles LECT2 in immune diseases

### 4.1 Tumor immunity

LECT2 functions as a tumor suppressor in many cancers (5, 46, 48, 51–53). Apart from directly interacting with cancer cells, LECT2 also modulates cancer progression *via* the tumor immune microenvironment (TIM) (51, 52). In hepatocellular carcinoma (HCC), LECT2 expression is negatively associated with the immune infiltration of monocyte, B cells, neutrophil, and myeloid dendritic cells and positively associated with hematopoietic stem cells and CD8 naive T cells. In addition, the LECT2 level is also negatively associated with multiple immune checkpoint molecules and HLA genes (51). Moreover, LECT2 is documented to prevent the recruitment of inflammatory monocytes and the acquisition of their immunosuppressive properties, and it has the ability to inhibit the EMT response and angiogenesis processes in β-catenin-activated hepatocytes (52). An oncogenic β-catenin-triggering inflammatory tumor microenvironment is indispensable for the aggressiveness of HCC in mice, and LECT2 inhibits HCC progression by blocking β-catenin-induced inflammation by interconnecting with invariant NKT (iNKT) cells (54). It has been shown that during intestinal tumorigenesis, compared to wild-type mice, LECT2-deficient mice exhibited a reduced overall survival and a significantly increased number of adenomas in the small intestine with increased severity (53). Further analysis showed that the homozygous loss of *Lect2* promoted intestinal tumorigenesis by changing the tumor microenvironment, indicated by altering the balance of pro- and anti-inflammatory cytokines and key regulators of the T-cell lineage in the Wnt-activated colorectal cancer model. All the results show that LECT2 is a potential anti-tumor cytokine for cancer therapy.

### 4.2 Non-alcoholic fatty liver disease

Non-alcoholic fatty liver disease (NAFLD) consists of a series of liver disorders ranging from hepatic steatosis to non-alcoholic steatohepatitis (NASH) and ultimately may lead to cirrhosis; its inflammatory responses are becoming the leading cause of liver-related morbidity and mortality worldwide (55). LECT2 is involved in all almost stages of NAFLD and is a potential diagnosis marker for this disease (7, 8, 25, 40, 56). In NAFLD, activating transcription factor 4 (ATF4) contributes to the upregulation of LECT2 transcription by binding to the *LECT2* gene promoter under ER stress response (56). LECT2 is also found to promote liver steatosis by shifting the liver residual macrophage to the M1-like phenotype and to contribute to the development of liver inflammation *via* JNK-mediated signaling in NASH (8). Another study also found that LECT2 induces the development of NAFLD by mediating the phosphorylation level of STAT-1 and the expression of its downstream genes cluster of differentiation 36 (Cd36), chemokine (C-X-C motif), ligand 10 (Cxcl10), and unc-51-like autophagy-activating kinase 1 (Ulk1) (7). In addition, LECT2 is also reported as a non-invasive diagnostic factor for alcohol-induced liver cirrhosis (9).

### 4.3 Acute liver injury

Acute liver injury (ALI) is commonly caused by bacterial endotoxin/lipopolysaccharide (LPS) or drug overdose; it causes a systemic inflammatory response syndrome that is clinically much like sepsis (57, 58). In ALI mouse models, LECT2 was upregulated, and LECT2-KO mice more significantly reduced liver injuries than wild-type mice. Another study found that LECT2 knockdown alleviates liver injuries by regulating monocyte/macrophage chemotaxis (59). This result suggests that LECT2 might be used as a therapeutic target for ALI.

### 4.4 Sepsis

Bacterial or viral infection is one of the main causes of sepsis (60–62). LECT2 was firstly found to be associated with bacterial and viral infection in multiple teleosts (63–66). the anti-bacterial/anti-viral roles of LECT2 have also been verified in Aves (67, 68), mammals (22, 69), and human beings (23, 70). In vertebrates, LECT2 was firstly considered to exhibit its anti-bacterial/anti-viral activities by activating immune cells (macrophages, heterophils, and lymphocytes) (22, 68, 71) and downregulating pro-inflammatory factors such as TNF-α and IL-6 (22, 24). Several studies have revealed that LECT2 relieves both bacteria- and virus- induced sepsis in different mechanisms (22, 42, 70). In viral sepsis, LECT2 promotes retinoic acid-inducible gene I (RIG-I)-mediated anti-virus immunity by interacting with MET receptor, and this process can be antagonized by an original MET ligand hepatocyte growth factor (HGF) (42). For bacterial sepsis, LECT2 enhances the bactericidal activity of macrophages by inducing the phosphorylation of CD209a at its residue Ser28 and then leading to Raf-1 and NK-κB activation (22, 70). LECT2 has also been found to shift the development of pro-inflammatoryTh1/Th17 cells to anti-inflammatory Treg cells *via* the differentiation of bone marrow-derived dendritic cells (BMDCs) into dendritic cells, secretion of inflammatory cytokines, and differentiation of T cells after *Helicobacter pylori* infection in a CD209a receptor-dependent manner, suggesting that interrupting the LECT2-CD209a interaction may provide a promising target for *H. pylori* clearance (72). Moreover, LECT2 has direct anti-bacterial activity in teleosts, and this activity is conserved among vertebrates apart from humans (69). Interestingly, although LECT2 has two copies in teleosts, only LECT2-b exhibits direct anti-bacterial activity in grass carp. Fish LECT2-b not only exhibits conserved chemotactic and phagocytosis-stimulating activities but also kills Gram^-^ and Gram^+^ bacteria directly in a membrane-dependent and a non-membrane-dependent manner, respectively. Additionally, LECT2-b impedes bacterial adherence to epithelial cells by inducing agglutination, which is achieved by binding peptidoglycan and lipoteichoic acid. All these results suggest that LECT2 is a potential drug for sepsis treatment.

### 4.5 Atherosclerosis

Atherosclerosis is a chronic, multifocal, lipid-driven immunoinflammatory disease that occurs in medium-sized and large arteries (73). LECT2 has been found to be a potential diagnosis biomarker for atherosclerosis and correlated with the developmental stage of atherosclerosis (25, 27). Another study found that LECT2 induces atherosclerotic inflammatory reaction *via* CD209a/JNK signaling in human endothelial cells (26). He et al. found that LECT2 administration reduces the concentrations of serum total cholesterol and low-density lipoprotein and the size of atherosclerotic lesions and thus impedes the progression of atherosclerosis (13).

### 4.6 Rheumatoid arthritis

Rheumatoid arthritis (RA) is a systemic autoimmune arthropathy and is characterized by a failure of inflammation to resolve automatically (74). Several studies have shown that LECT2 is a potential biomarker for RA diagnosis (30–33). Multiple clinical statistics have considered that the Val58Ile polymorphism of LECT2 is associated with the joint destruction in RA (32, 33). In a mouse arthritis model, LECT2^-/-^ mice exhibited more severe arthritic symptoms than the wild-type controls, which were indicated by LECT2^-/-^ mice having more severe inflammation and erosion of cartilage and bone. It was also found that exogenous expression of LECT2 can alleviate arthritis symptoms in LECT2 knockout mice (31), which strongly suggests that LECT2 treatment might be a potential strategy against inflammatory arthritis such as RA.

### 4.7 Osteoporosis

Osteoporosis (OS) is caused by the imbalance in the ratio between osteoblasts and osteoclasts, which is closely associated with osteogenic differentiation (OD) (28). LECT2 is low expression in mesenchymal stem cells (MSCs) with OD, and it inhibits OD in MSCs by inactivating the Wnt/β-catenin pathway. LECT2 is also found to play a role in the upregulation in serum of osteoporosis patients, is positively correlated with their bone loss, and is a potential biomarker for osteoporosis diagnosis (29). All these results suggest that LECT2 is a potential diagnosis and therapeutic target for osteoporosis.

### 4.8 Allergic diseases

Allergic diseases such as atopic dermatitis and parasitic infection are severe systemic hypersensitivity reactions that are rapid in onset and usually associated with skin and immune system changes, which seriously affect a patient’s health and quality of life (75, 76). Jeronimo et al. found that LECT2 is involved in modulating delayed-type hypersensitivity responses resulting from *Leishmania chagasi* infection, but its potential mechanism is unclear (77). In addition, Zhao et al. revealed that serum LECT2 is positively correlated with atopic dermatitis and its severity (35), but the mechanisms of how LECT2 mediates the progression of atopic dermatitis remain unknown.

## 5 Concluding remarks and perspectives

In its structure, LECT2 has high similarity among different vertebrates, indicated by their high conservation of three disulfide bonds and three metal-binding sites. The homology of LECT2 between different species provides the possibility to study its functions using comparative immunological methods. Currently, two of the five reported ligand proteins (CD209a and Trf) of LECT2 were firstly identified by our group in teleosts using the yeast two-hybrid system (45, 64), and the two interactions were further verified by our and the other groups. The LECT2-CD209a interaction was found to mediate bacterial clearance and obesity and drive the expansion and mobility of HSCs by modulating the macrophages and osteolineage cells (41). For LECT2-Trf interaction, although this protein–protein interaction (PPI) exists from teleosts to mice (45), its specific pathophysiologic functions are still unknown. Additionally, the complex assembled by LECT2, L1CAM, MNR-1, and DMA-1 is indispensable for the growth of dendritic terminal branches in *C. elegans* (44). Although all the homologous components of this complex are present in vertebrates, whether this complex exists in vertebrates and its potential roles remain unknown. These discoveries of LECT2 receptors further verify the feasibility of the digesting functions of novel proteins by comparative immunology.

Mounting evidence supports that LECT2 has versatile roles in immune diseases. It attenuates tumorigenesis by modulating TIM (51, 52); modulates inflammatory responses in several tissues such as liver (7, 8, 25, 40, 56, 59), bone marrow (29), joints (30–33), and blood vessels (13, 25–27); alleviates bacteria/virus-induced sepsis (22, 23, 42, 67–71); and accelerates the progression of allergic diseases (35, 77). It also mediates the Wnt/β-catenin pathway to regulate osteogenic differentiation of MSCs (28). The interaction of LECT2 with CD209a promotes the proliferation of HSCs in the bone marrow and mobilization to the blood, and it also regulates HSC homeostasis by affecting the expression of TNFα in macrophages and osteoblasts (41). Given the multifunctionality of LECT2 and its theranostical application in multiple immune-related diseases, there are many areas that are worth further investigating. Firstly, many of the LECT2-mediated pathophysiologic roles interplay with each other, and it is imperative to investigate whether and how LECT2 modulates the crosstalk among different immune diseases. For example, LECT2 is involved in the development of several liver immune diseases such as NAFLD, insulin resistance, liver regeneration, and HCC. LECT2 is the common mediator for them, and these liver immune diseases can be interchangeable or occur simultaneously. However, no related literature describes the role of LECT2 in their crosstalk. Secondly, considering the broad spectrum of LECT2-mediated liver immune diseases, it is theoretically feasible to construct an algorithm using LECT2 levels to predicate the progression of these diseases. Thirdly, LECT2 has been found to promote the progress of several immune diseases and is considered as a therapeutic target, but no agent with activity that reduces LECT2 levels has been identified for the moment. Further studies are needed to screen/identify agents with functions that lower LECT2 levels. Finally, the potentially clinical applications of LECT2 in immune diseases have been verified in a mouse model. However, further study is still imperative to shed light on their action mechanisms to avoid unpredicted risks before LECT2 is used clinically in humans. For example, recombinant LECT2 (rLECT2) administration was found to alleviate the sepsis induced by bacteria and virus in a mouse model, and LECT2 is also negatively associated with sepsis in humans, but there is a dearth of studies about whether LECT2 has similar mechanisms and efficacy. Therefore, there remains a need for studies focusing on the action mechanism and clinical applications of rLECT2 in humans. Further exploration of the role of LECT2 in varieties of immune diseases and its correlation with clinical immune-related diseases will advance the development of LECT2 as an appealing theranostical target for immune diseases.

## Author contributions

All authors listed have made a substantial, direct, and intellectual contribution to the work, and approved it for publication.

## References

[1] Yamagoe S Yamakawa Y Matsuo Y Minowada J Mizuno S Suzuki K. Purification and primary amino acid sequence of a novel neutrophil chemotactic factor LECT2. Immunol Lett (1996) 52(1):9–13. doi: 10.1016/0165-2478(96)02572-2 8877413

[2] Hiraki Y Inoue H Kondo J Kamizono A Yoshitake Y Shukunami C. A novel growth-promoting factor derived from fetal bovine cartilage, chondromodulin II: Purification and amino acid sequence. J Biol Chem (1996) 271(37):22657–62. doi: 10.1074/jbc.271.37.22657 8798437

[3] Zhu S Qiu H Bennett S Kuek V Rosen V Xu H. Chondromodulin-1 in health, osteoarthritis, cancer, and heart disease. Cell Mol Life Sci (2019) 76(22):4493–502. doi: 10.1007/s00018-019-03225-y PMC6841647 31317206

[4] Shukunami C Hiraki Y. Role of cartilage-derived anti-angiogenic factor, chondromodulin-I, during endochondral bone formation. Osteoarthr Cartil (2001) 9:S91–101. doi: 10.1053/joca.2001.0450 11680695

[5] Chen C-K Yu W-H Cheng T-Y Chen M-W Su C-Y Yang Y-C. Inhibition of VEGF165/VEGFR2-dependent signaling by LECT2 suppresses hepatocellular carcinoma angiogenesis. Sci Rep (2016) 6(1):1–12. doi: 10.1038/srep31398 27507763 PMC4979047

[6] Xu M Xu H Lin Y Sun X Wang L Fang Z. LECT2, a ligand for Tie1, plays a crucial role in liver fibrogenesis. Cell (2019) 178(6):1478–1492.e20. doi: 10.1016/j.cell.2019.07.021 31474362

[7] Wang J Chen Y Pan R Wu C Chen S Li L. Leukocyte cell-derived chemotaxin 2 promotes the development of nonalcoholic fatty liver disease through STAT-1 pathway in mice. Liver Int (2021) 41(4):777–87. doi: 10.1111/liv.14816 33555112

[8] Takata N Ishii K Takayama H Nagashimada M Kamoshita K Tanaka T. LECT2 as a hepatokine links liver steatosis to inflammation *via* activating tissue macrophages in NASH. Sci Rep (2021) 11(1):555. doi: 10.1038/s41598-020-80689-0 33436955 PMC7804418

[9] Sak J Prystupa A Kiciński P Luchowska-Kocot D Kurys-Denis E Bis-Wencel H. Leukocyte cell-derived chemotaxin-2 and fibroblast growth factor 21 in alcohol-induced liver cirrhosis. World J Hepatol (2021) 13(12):2071–80. doi: 10.4254/wjh.v13.i12.2071 PMC8727211 35070009

[10] Kim J Lee S Kim D Lee E Park C Choe H. Adipose tissue LECT2 expression is associated with obesity and insulin resistance in Korean women. Obes (Silver Spring Md.) (2022) 30(7):1430–41. doi: 10.1002/oby.23445 35722819

[11] Qin Y Xiao K Zhong Z Zhao Y Yu T Sun X. LECT2 ameliorates blood-retinal barrier impairment secondary to diabetes *Via* activation of the Tie2/Akt/mTOR signaling pathway. Invest Ophthalmol Vis Sci (2022) 63(3):7. doi: 10.1167/iovs.63.3.7 PMC8934553 35262733

[12] Misu H. Identification of hepatokines involved in pathology of type 2 diabetes and obesity. Endocrine J (2019) 66(8):659–62. doi: 10.1507/endocrj.EJ19-0255 31366824

[13] He W Dai T Chen J Wang J. Leukocyte cell-derived chemotaxin 2 inhibits development of atherosclerosis in mice. Zoological Res (2019) 40(4):317–23. doi: 10.24272/j.issn.2095-8137.2019.030 PMC6680125 31310065

[14] Chikamoto K Misu H Takayama H Kikuchi A Ishii K Lan F. Rapid response of the steatosis-sensing hepatokine LECT2 during diet-induced weight cycling in mice. Biochem Biophys Res Commun (2016) 478(3):1310–6. doi: 10.1016/j.bbrc.2016.08.117 27562717

[15] Willis S Sargeant J Yates T Takamura T Takayama H Gupta V. High-fat feeding increases circulating FGF21, LECT2, and fetuin-a in healthy men. J Nutr (2020) 150(5):1076–85. doi: 10.1093/jn/nxz333 31919514

[16] Xie Y Fan K Guan S Hu Y Gao Y Zhou W. LECT2: A pleiotropic and promising hepatokine, from bench to bedside. J Cell Mol Med (2022) 26(13):3598–607. doi: 10.1111/jcmm.17407 PMC9258709 35656863

[17] Mann B Bhandohal J Cobos E Chitturi C Eppanapally S. LECT-2 amyloidosis: What do we know? J Invest Med (2022) 70(2):348–53. doi: 10.1136/jim-2021-002149 34848562

[18] Zhu S Bennett S Li Y Liu M Xu J. The molecular structure and role of LECT2 or CHM-II in arthritis, cancer, and other diseases. J Cell Physiol (2022) 237(1):480–8. doi: 10.1002/jcp.30593 34550600

[19] Benson M. LECT2 amyloidosis. Kidney Int (2010) 77(9):757–9. doi: 10.1038/ki.2010.18 20393490

[20] Benson M James S Scott K Liepnieks J Kluve-Beckerman B. Leukocyte chemotactic factor 2: A novel renal amyloid protein. Kidney Int (2008) 74(2):218–22. doi: 10.1038/ki.2008.152 18449172

[21] Larsen C Walker P Weiss D Solomon A. Prevalence and morphology of leukocyte chemotactic factor 2-associated amyloid in renal biopsies. Kidney Int (2010) 77(9):816–9. doi: 10.1038/ki.2010.9 PMC11181467 20182418

[22] Lu X Chen J Yu C Shi Y He Y Zhang R. LECT2 protects mice against bacterial sepsis by activating macrophages *via* the CD209a receptor. J Exp Med (2013) 210(1):5–13. doi: 10.1084/jem.20121466 23254286 PMC3549712

[23] Ando K Kato H Kotani T Ozaki M Arimura Y Yagi J. Plasma leukocyte cell-derived chemotaxin 2 is associated with the severity of systemic inflammation in patients with sepsis. Microbiol Immunol (2012) 56(10):708–18. doi: 10.1111/j.1348-0421.2012.00488.x 22725643

[24] Dang M Kato H Ueshiba H Omori-Miyake M Yamagoe S Ando K. Possible role of LECT2 as an intrinsic regulatory factor in SEA-induced toxicity in d-galactosamine-sensitized mice. Clin Immunol (Orlando Fla.) (2010) 137(3):311–21. doi: 10.1016/j.clim.2010.08.002 20805039

[25] Yoo H Hwang S Choi J Lee H Chung H Seo J. Association of leukocyte cell-derived chemotaxin 2 (LECT2) with NAFLD, metabolic syndrome, and atherosclerosis. PloS One (2017) 12(4):e0174717. doi: 10.1371/journal.pone.0174717 28376109 PMC5380318

[26] Hwang H Jung T Hong H Seo J Kim S Kim N. LECT2 induces atherosclerotic inflammatory reaction *via* CD209 receptor-mediated JNK phosphorylation in human endothelial cells. Metabolism: Clin Exp (2015) 64(9):1175–82. doi: 10.1016/j.metabol.2015.06.001 26123523

[27] Sonmez F Yildiz P Akhtar M Aydin C Sonmez O Ay N. New markers in atherosclerosis: Thrombospondin-2 (THBS-2) and leukocyte cell-derived chemotaxin-2 (LECT-2); An immunohistochemical study. Med Sci Monit (2016) 22:5234–9. doi: 10.12659/MSM.898889 PMC5221427 28039493

[28] Xu Z He J Zhou X Zhang Y Huang Y Xu N. Down-regulation of LECT2 promotes osteogenic differentiation of MSCs *via* activating wnt/β-catenin pathway. Biomed Pharmacother (2020) 130:110593. doi: 10.1016/j.biopha.2020.110593 32763823

[29] Wang Q Xu F Chen J Xie Y Xu S He W. Serum leukocyte cell-derived chemotaxin 2 (LECT2) level is associated with osteoporosis. Lab Med (2023) 54(1):106–11. doi: 10.1093/labmed/lmac080 35976970

[30] Ikeda D Ageta H Tsuchida K Yamada H. iTRAQ-based proteomics reveals novel biomarkers of osteoarthritis. Biomarkers (2013) 18(7):565–72. doi: 10.3109/1354750X.2013.810667 PMC3836424 23937207

[31] Okumura A Saito T Otani I Kojima K Yamada Y Ishida-Okawara A. Suppressive role of leukocyte cell-derived chemotaxin 2 in mouse anti-type II collagen antibody-induced arthritis. Arthritis Rheumatism (2008) 58(2):413–21. doi: 10.1002/art.23215 18240267

[32] Graessler J Verlohren M Graessler A Zeissig A Kuhlisch E Kopprasch S. Association of chondromodulin-II Val58Ile polymorphism with radiographic joint destruction in rheumatoid arthritis. J Rheumatol (2005) 32(9):1654–61. 16142856

[33] Kameoka Y Yamagoe S Hatano Y Kasama T Suzuki K. Val58Ile polymorphism of the neutrophil chemoattractant LECT2 and rheumatoid arthritis in the Japanese population. Arthritis rheumatism (2000) 43(6):1419–20. doi: 10.1002/1529-0131(200006)43:6<1419::AID-ANR28>3.0.CO;2-I 10857804

[34] Lan F Misu H Chikamoto K Takayama H Kikuchi A Mohri K. LECT2 functions as a hepatokine that links obesity to skeletal muscle insulin resistance. Diabetes (2014) 63(5):1649–64. doi: 10.2337/db13-0728 24478397

[35] Zhao K Xu F Jiang X Chen J Zhu X Zhou Q. Serum leukocyte cell-derived chemotaxin 2 level is associated with atopic dermatitis patients. Ann Palliat Med (2021) 10(10):11006–12. doi: 10.21037/apm-21-2690 34763463

[36] Yamagoe S Kameoka Y Hashimoto K Mizuno S Suzuki K. Molecular cloning, structural characterization, and chromosomal mapping of the human LECT2 gene. Genomics (1998) 48(3):324–9. doi: 10.1006/geno.1997.5198 9545637

[37] Zheng H Miyakawa T Sawano Y Asano A Okumura A Yamagoe S. Crystal structure of human leukocyte cell-derived chemotaxin 2 (LECT2) reveals a mechanistic basis of functional evolution in a mammalian protein with an M23 metalloendopeptidase fold. J Biol Chem (2016) 291(33):17133–42. doi: 10.1074/jbc.M116.720375 PMC5016117 27334921

[38] Okumura A Suzuki T Miyatake H Okabe T Hashimoto Y Miyakawa T. Leukocyte cell-derived chemotaxin 2 is a zinc-binding protein. FEBS Lett (2013) 587(5):404–9. doi: 10.1016/j.febslet.2013.01.025 23352894

[39] Nagai H Hamada T Uchida T Yamagoe S Suzuki K. Systemic expression of a newly recognized protein, LECT2, in the human body. Pathol Int (1998) 48(11):882–6. doi: 10.1111/j.1440-1827.1998.tb03855.x 9832057

[40] Berthou F Sobolewski C Abegg D Fournier M Maeder C Dolicka D. Hepatic PTEN signaling regulates systemic metabolic homeostasis through hepatokines-mediated liver-to-Peripheral organs crosstalk. Int J Mol Sci (2022) 23(7):3959. doi: 10.3390/ijms23073959 35409319 PMC8999584

[41] Lu X Chen Q Rong Y Yang G Li C Xu N. LECT2 drives haematopoietic stem cell expansion and mobilization *via* regulating the macrophages and osteolineage cells. Nat Commun (2016) 7:12719. doi: 10.1038/ncomms12719 27596364 PMC5025878

[42] Shirasaki T Yamagoe S Shimakami T Murai K Imamura R Ishii K. Leukocyte cell-derived chemotaxin 2 is an antiviral regulator acting through the proto-oncogene MET. Nat Commun (2022) 13(1):3176. doi: 10.1038/s41467-022-30879-3 35676290 PMC9177837

[43] Chen C Yang C Hua K Hua K Ho M Johansson G. Leukocyte cell-derived chemotaxin 2 antagonizes MET receptor activation to suppress hepatocellular carcinoma vascular invasion by protein tyrosine phosphatase 1B recruitment. Hepatol (Baltimore Md.) (2014) 59(3):974–85. doi: 10.1002/hep.26738 24114941

[44] Zou W Shen A Dong X Tugizova M Xiang Y Shen K. A multi-protein receptor-ligand complex underlies combinatorial dendrite guidance choices in *C. elegance* . eLife (2016) 5:e18345. doi: 10.7554/eLife.18345 27705746 PMC5079751

[45] Chen J Yang H Shi Y Li M. An interaction between leukocyte cell-derived chemotaxin 2 and transferrin of ayu, plecoglossus altivelis. Fish Shellfish Immunol (2009) 26(3):536–42. doi: 10.1016/j.fsi.2009.02.010 19223015

[46] Chu T Ko C Tai P Chang Y Huang C Wu T. Leukocyte cell-derived chemotaxin 2 regulates epithelial-mesenchymal transition and cancer stemness in hepatocellular carcinoma. J Biol Chem (2022) 298(10):102442. doi: 10.1016/j.jbc.2022.102442 36055405 PMC9530851

[47] Li X Lin P Tao Y Jiang X Li T Wang Y. LECT 2 antagonizes FOXM1 signaling *via* inhibiting MET to retard PDAC progression. Front Cell Dev Biol (2021) 9:661122. doi: 10.3389/fcell.2021.661122 33937262 PMC8082113

[48] Hung W Chang J Cheng Y Chen C Chen J Hua K. Leukocyte cell-derived chemotaxin 2 retards non-small cell lung cancer progression through antagonizing MET and EGFR activities. Cell Physiol Biochem (2018) 51(1):337–55. doi: 10.1159/000495233 30453282

[49] Woo K Baldwin H. Role of Tie1 in shear stress and atherosclerosis. Trends Cardiovasc Med (2011) 21(4):118–23. doi: 10.1016/j.tcm.2012.03.009 PMC3382080 22681967

[50] Kawabata H. Transferrin and transferrin receptors update. Free Radical Biol Med (2019) 133:46–54. doi: 10.1016/j.freeradbiomed.2018.06.037 29969719

[51] Qin J Sun W Zhang H Wu Z Shen J Wang W. Prognostic value of LECT2 and relevance to immune infiltration in hepatocellular carcinoma. Front Genet (2022) 13:951077. doi: 10.3389/fgene.2022.951077 36160006 PMC9500357

[52] L’Hermitte A Pham S Cadoux M Couchy G Caruso S Anson M. Lect2 controls inflammatory monocytes to constrain the growth and progression of hepatocellular carcinoma. Hepatol (Baltimore Md.) (2019) 69(1):160–78. doi: 10.1002/hep.30140 30070727

[53] Greenow K Zverev M May S Kendrick H Williams G Phesse T. Lect2 deficiency is characterised by altered cytokine levels and promotion of intestinal tumourigenesis. Oncotarget (2018) 9(92):36430–43. doi: 10.18632/oncotarget.26335 PMC6284865 30559928

[54] Anson M Crain-Denoyelle A Baud V Chereau F Gougelet A Terris B. Oncogenic β-catenin triggers an inflammatory response that determines the aggressiveness of hepatocellular carcinoma in mice. J Clin Invest (2012) 122(2):586–99. doi: 10.1172/JCI43937 PMC3266772 22251704

[55] Powell E Wong V Rinella M. Non-alcoholic fatty liver disease. Lancet (London England) (2021) 397(10290):2212–24. doi: 10.1016/S0140-6736(20)32511-3 33894145

[56] Park C Lee S Kim J Kim D Choe H Jeong J. Endoplasmic reticulum stress increases LECT2 expression *via* ATF4. Biochem Biophys Res Commun (2021) 585:169–76. doi: 10.1016/j.bbrc.2021.11.038 34808500

[57] Zhang Y Xue W Zhang W Yuan Y Zhu X Wang Q. Histone methyltransferase G9a protects against acute liver injury through GSTP1. Cell Death Differ (2020) 27(4):1243–58. doi: 10.1038/s41418-019-0412-8 PMC7206029 31515511

[58] Zhang Q Raoof M Chen Y Sumi Y Sursal T Junger W. Circulating mitochondrial DAMPs cause inflammatory responses to injury. Nature (2010) 464(7285):104–7. doi: 10.1038/nature08780 PMC2843437 20203610

[59] Xie Y Zhong K Hu Y Xi Y Guan S Xu M. Liver infiltration of multiple immune cells during the process of acute liver injury and repair. World J Gastroenterol (2022) 28(46):6537–50. doi: 10.3748/wjg.v28.i46.6537 PMC9782841 36569272

[60] Delano M Ward P. The immune system’s role in sepsis progression, resolution, and long-term outcome. Immunol Rev (2016) 274(1):330–53. doi: 10.1111/imr.12499 PMC5111634 27782333

[61] Gautier T Deckert V Nguyen M Desrumaux C Masson D Lagrost L. New therapeutic horizons for plasma phospholipid transfer protein (PLTP): Targeting endotoxemia, infection and sepsis. Pharmacol Ther (2022) 236:108105. doi: 10.1016/j.pharmthera.2021.108105 34974028

[62] Cole J Morris P Dickman M Dockrell D. The therapeutic potential of epigenetic manipulation during infectious diseases. Pharmacol Ther (2016) 167:85–99. doi: 10.1016/j.pharmthera.2016.07.013 27519803 PMC5109899

[63] Lin B Chen S Cao Z Lin Y Mo D Zhang H. Acute phase response in zebrafish upon aeromonas salmonicida and staphylococcus aureus infection: Striking similarities and obvious differences with mammals. Mol Immunol (2007) 44(4):295–301. doi: 10.1016/j.molimm.2006.03.001 16630661

[64] Li M Chen J Shi Y. Molecular cloning of leucocyte cell-derived chemotaxin-2 gene in croceine croaker (Pseudosciaena crocea). Fish Shellfish Immunol (2008) 24(2):252–6. doi: 10.1016/j.fsi.2007.09.003 18155922

[65] Hansen J Woodson J Hershberger P Grady C Gregg J Purcell M. Induction of anti-viral genes during acute infection with viral hemorrhagic septicemia virus (VHSV) genogroup IVa in pacific herring (Clupea pallasii). Fish Shellfish Immunol (2012) 32(2):259–67. doi: 10.1016/j.fsi.2011.11.010 22155011

[66] Chen J Chen Q Lu X Li C. LECT2 improves the outcomes in ayu with vibrio anguillarum infection *via* monocytes/macrophages. Fish Shellfish Immunol (2014) 41(2):586–92. doi: 10.1016/j.fsi.2014.10.012 25462453

[67] Xu Q Chen Y Tong Y Huang Z Zhao W Duan X. Identification and expression analysis of the leukocyte cell-derived chemotaxin-2 (LECT2) gene in duck (Anas platyrhynchos). Gene (2014) 533(1):280–5. doi: 10.1016/j.gene.2013.09.047 24076354

[68] Sekelova Z Stepanova H Polansky O Varmuzova K Faldynova M Fedr R. Differential protein expression in chicken macrophages and heterophils *in vivo* following infection with salmonella enteritidis. Vet Res (2017) 48(1):35. doi: 10.1186/s13567-017-0439-0 28623956 PMC5473982

[69] Hu Y Ma Z Wu C Wang J Zhang Y Zhang X. LECT2 is a novel antibacterial protein in vertebrates. J Immunol (Baltimore Md.: 1950) (2022) 208(8):2037–53. doi: 10.4049/jimmunol.2100812 35365566

[70] Shen H Li L Chen Q He Y Yu C Chu C. LECT2 association with macrophage-mediated killing of helicobacter pylori by activating NF-κB and nitric oxide production. Genet Mol Res: GMR (2016) 15(4):gmr15048889. doi: 10.4238/gmr15048889 27813598

[71] Wang Z Lu J Li C Li Q Pang Y. Characterization of the LECT2 gene and its protective effects against microbial infection *via* large lymphocytes in lampetra japonica. Dev Comp Immunol (2018) 79:75–85. doi: 10.1016/j.dci.2017.09.018 29056545

[72] Zhang X Sun K Tang C Cen L Li S Zhu W. LECT2 modulates dendritic cell function after helicobacter pylori infection *via* the CD209a receptor. J Gastroenterol Hepatol (2023). doi: 10.1111/jgh.16138 36740832

[73] Bäck M Yurdagul A Tabas I Öörni K Kovanen P. Inflammation and its resolution in atherosclerosis: Mediators and therapeutic opportunities. Nat Rev Cardiol (2019) 16(7):389–406. doi: 10.1038/s41569-019-0169-2 30846875 PMC6727648

[74] Finckh A Gilbert B Hodkinson B Bae S Thomas R Deane K. Global epidemiology of rheumatoid arthritis, nature reviews. Rheumatology (2022) 18(10):591–602. doi: 10.1038/s41584-022-00827-y 36068354

[75] Bieber T. Atopic dermatitis: an expanding therapeutic pipeline for a complex disease, nature reviews. Drug Discov (2022) 21(1):21–40. doi: 10.1038/s41573-021-00266-6 PMC8377708 34417579

[76] Cruz A Cooper P Figueiredo C Alcantara-Neves N Rodrigues L Barreto M. Global issues in allergy and immunology: Parasitic infections and allergy. J Allergy Clin Immunol (2017) 140(5):1217–28. doi: 10.1016/j.jaci.2017.09.005 29108604

[77] Jeronimo S Holst A Jamieson S Francis R Martins D Bezerra F. Genes at human chromosome 5q31.1 regulate delayed-type hypersensitivity responses associated with leishmania chagasi infection. Genes Immun (2007) 8(7):539–51. doi: 10.1038/sj.gene.6364422 PMC2435172 17713557

